# Personalized messenger RNA therapy for melanoma and the challenge of global implementation

**DOI:** 10.1016/j.jdin.2026.08.005

**Published:** 2026-08-28

**Authors:** Umayr R. Shaikh, Jessica Gai, Sakshi Chopra, Chloe Bush, Harold William Higgins, Jane M. Grant-Kels

**Affiliations:** a Georgetown University School of Medicine, Washington, DC; b Department of Dermatology, University of Pennsylvania, Philadelphia, Pennsylvania; c Department of Dermatology, University of Connecticut, Farmington, Connecticut; d University of Florida Health Dermatology, Gainesville, Florida

**Keywords:** melanoma, neoantigen, personalized medicine, immunotherapy, cancer vaccine, global health

*To the Editor:*

On August 19, 2026, positive topline results were announced from INTerpath-001, the first phase III trial to demonstrate benefit from an individualized neoantigen therapy. Among 1137 patients with completely resected stage IIB-IV cutaneous melanoma, intismeran autogene plus pembrolizumab was reported to produce statistically significant and clinically meaningful improvements in recurrence-free and distant metastasis-free survival compared with pembrolizumab alone.^1^ Although full results await presentation and peer review, and overall survival remains under evaluation, the announcement represents a milestone in melanoma therapeutics.

Intismeran is not a preventive vaccine in the conventional sense. Tumor-derived mutations are identified and prioritized to generate patient-specific messenger RNA encoding up to 34 neoantigens, with the goal of eliciting T-cell responses against an individual tumor’s mutational repertoire.^1^ This concept arose from foundational studies demonstrating that personalized melanoma vaccines could induce polyclonal neoantigen-specific T-cell responses and recognize autologous tumor cells.^2^^,^^3^ Less than a decade later, personalized neoantigen therapy has progressed from first-in-human feasibility to phase III efficacy.

The trajectory is compelling. In the randomized phase IIb KEYNOTE-942 trial, intismeran plus pembrolizumab improved recurrence-free survival compared with pembrolizumab alone, with an 18-month recurrence-free survival of 79% versus 62%.^4^ After 5 years of follow-up, this benefit remained durable because the combination was associated with lower risks of recurrence or death (hazard ratio, 0.51) and distant metastasis or death (hazard ratio, 0.41), without new safety signals.^5^

Yet, the same personalization underlying this advance may also determine how broadly its benefits can be realized. Individualized neoantigen therapy requires adequate tumor tissue, genomic characterization, computational neoantigen selection, bespoke manufacturing, and coordinated administration with systemic immunotherapy.^1^, ^2^, ^3^ Early personalized melanoma vaccine studies demonstrated the feasibility of this approach but also illustrated its logistical intensity, with individualized production occurring over weeks to months.^2^^,^^3^ Contemporary processes have evolved, but scalability, manufacturing capacity, turnaround time, cost, and infrastructure will become increasingly consequential if regulatory approvals follow.

These challenges are particularly important globally. Precision therapies may magnify differences between health systems with the infrastructure to support molecular diagnostics, specimen transport, individualized manufacturing, and checkpoint inhibition and those without these capabilities. Access may therefore depend not only on regulatory approval or drug availability but also on whether patients can move efficiently through each step from tissue acquisition to sequencing, production, delivery, and oncologic follow-up. The next measure of success cannot be efficacy alone. Trials and implementation studies should examine geographic representation, tissue-to-treatment intervals, manufacturing success, affordability, and access across diverse health systems.

Dermatologists will remain central throughout this transition, from diagnosis and tissue acquisition to patient counseling and longitudinal surveillance. They will also be positioned to clarify an important distinction for patients: this is a personalized therapeutic cancer vaccine, and it is not a vaccine to prevent melanoma. As these therapies move toward clinical use, dermatologists will need familiarity with their mechanism, indications, limitations, and implications for multidisciplinary care.

Personalized messenger RNA therapy may represent a new chapter in melanoma treatment. Ensuring that personalization does not become synonymous with limited accessibility, and that cost does not become an impediment to distributive justice, should define the chapter that follows.

## Declaration of Generative AI and AI-Assisted Technologies in the Writing Process

Not used.

## Conflicts of interest

None disclosed.

## References

[1] Merck & Co, Inc, Moderna, Inc (2026). Merck and Moderna announce phase 3 INTerpath-001 trial of intismeran autogene plus KEYTRUDA met endpoints of recurrence-free survival (RFS) and distant metastasis-free survival (DMFS) in patients with completely resected stage IIB-IV melanoma. https://www.merck.com/news/merck-and-moderna-announce-phase-3-interpath-001-trial-of-intismeran-autogene-plus-keytruda-met-endpoints-of-recurrence-free-survival-rfs-and-distant-metastasis-free-survival-dmfs-in-patient/.

[2] Shin U., Derhovanessian E., Miller M. (2017). Personalized RNA mutasome vaccines mobilize poly-specific therapeutic immunity against cancer. Nature.

[3] Ott P.A., Hu Z., Keskin D.B. (2017). An immunogenic personal neoantigen vaccine for patients with melanoma. Nature.

[4] Weber J.S., Carlino M.S., Khattak A. (2024). Individualised neoantigen therapy mRNA-4157 (V940) plus pembrolizumab versus pembrolizumab monotherapy in resected melanoma (KEYNOTE-942): a randomised, phase 2b study. Lancet.

[5] Khattak A., Carlino M.S., Meniawy T. (Published online June 1, 2026). Intismeran autogene plus pembrolizumab versus pembrolizumab alone in high-risk resected melanoma: 5-year update of the randomized phase IIb KEYNOTE-942 study. J Clin Oncol.

